# Extracellular Localization of the Diterpene Sclareol in Clary Sage (*Salvia sclarea* L., *Lamiaceae*)

**DOI:** 10.1371/journal.pone.0048253

**Published:** 2012-10-25

**Authors:** Jean-Claude Caissard, Thomas Olivier, Claire Delbecque, Sabine Palle, Pierre-Philippe Garry, Arthur Audran, Nadine Valot, Sandrine Moja, Florence Nicolé, Jean-Louis Magnard, Sylvain Legrand, Sylvie Baudino, Frédéric Jullien

**Affiliations:** 1 Laboratoire de Biotechnologies Végétales Appliquées aux Plantes Aromatiques et Médicinales, Université Jean Monnet, Université de Lyon, Saint-Etienne, France; 2 Laboratoire Hubert Curien, Université Jean Monnet, Université de Lyon, Saint-Etienne, France; 3 Bontoux S.A., Saint-Auban-sur-Ouvèze, France; 4 Centre de Microscopie Confocale Multiphotonique, Université Jean Monnet, Université de Lyon, Saint-Etienne, France; 5 Laboratoire Stress Abiotiques et Différenciation des Végétaux Cultivés, Université Lille Nord de France, Université Lille 1, Villeneuve d'Ascq, France; Lawrence Berkeley National Laboratory, United States of America

## Abstract

Sclareol is a high-value natural product obtained by solid/liquid extraction of clary sage (*Salvia sclarea* L.) inflorescences. Because processes of excretion and accumulation of this labdane diterpene are unknown, the aim of this work was to gain knowledge on its sites of accumulation *in planta*. Samples were collected *in natura* or during different steps of the industrial process of extraction (steam distillation and solid/liquid extraction). Samples were then analysed with a combination of complementary analytical techniques (gas chromatography coupled to a mass spectrometer, polarized light microscopy, environmental scanning electron microscopy, two-photon fluorescence microscopy, second harmonic generation microscopy). According to the literature, it is hypothesized that sclareol is localized in oil pockets of secretory trichomes. This study demonstrates that this is not the case and that sclareol accumulates in a crystalline epicuticular form, mostly on calyces.

## Introduction

Ambergris is a biogenic volatile organic compound with a sweet earthy aroma and a very high value. It is essentially used as a fixative in fragrance chemistry. It is a fatty secretion of the intestine of male sperm whales (*Physeter macrocephalus* L.) produced after a beak injury with a squid. Details about its maturation process are unknown and it is assumed that a freshly expelled viscous block of ambergris floats on the sea, loses some volatiles by evaporation, ages during a long period, and washes ashore as a 10 g to 1 kg lump.

Unfortunately, due to high demand and decrease in whale populations, ambergris has become a scarce product. As a consequence, chemists and perfumers have designed synthetic routes for the industrial production of (+/−)-norlabdane oxide, the most odorant molecule of ambergris. Most strategies of chemical synthesis are based on the hemisynthesis of (+/−)-norlabdane oxide from simple natural diterpenes or carotenoid derivatives such as nerolidol, β-ionone, thujone, manool and sclareol [1]–[4] and refs therein. This last starting substrate can be transformed via synthetic organic chemistry to sclareolide and then to (+/−)-norlabdane oxide by a two-step oxidation process and a reduction step respectively.

Sclareol can be extracted from inflorescences of *Salvia sclarea* L., a pluriannual herb from the *Lamiaceae* family, which is more commonly cultivated for its essential oil. The relatively easy farming of this herb and its high sclareol yield have encouraged clary sage producers to begin genetic improvement programs and expand clary sage plantations. However, the sclareol yield is very variable for reasons that are unknown but related to the different ability of clary sage inflorescences to accumulate sclareol during field production. Differences in sclareol yield are also related to the extraction process. According to an AFNOR directive (NFT 75–255) and industrials, essential oil obtained from 1,000 kg of dry straw contains 20 to 260 g (*i.e*. 0.002% to 0.026% yield) of sclareol. This should be compared to the 1.5% yield, which can be obtained by manufacturers when they use solid/liquid extraction (sclareol vs. organic solvent). The huge difference in yields is not explained.

In all members of the *Lamiaceae* that have been studied, essential oil accumulation takes place in subcuticular pockets of secretory trichomes, that are scattered over most of the plant epidermis from leaves to flower calyces and bracts [5]–[10]. In clary sage, essential oil is obtained by steam distillation of fresh inflorescences in full bloom and mostly contains mono- and sesquiterpenes [3], [10]–[13]. Even though clary sage essential oil contains some sclareol, the main site of its accumulation is unknown and cannot be assumed to be within or outside the glandular trichomes. The lack of information on the physiological aspects of sclareol accumulation within clary sage hampers efforts to improve both knowledge on diterpene biology and agronomic practices.

In this work, investigations on sclareol localization were undertaken at the cellular level with a combination of complementary techniques of microscopy (histochemistry; environmental scanning electron microscopy, ESEM; two-photon fluorescence microscopy, TPF microscopy; and second harmonic generation microscopy, SHG microscopy) and gas chromatography coupled to mass spectrometry (GC-MS).

## Results

### Tissue localization of sclareol in clary sage

**Figure 1:**
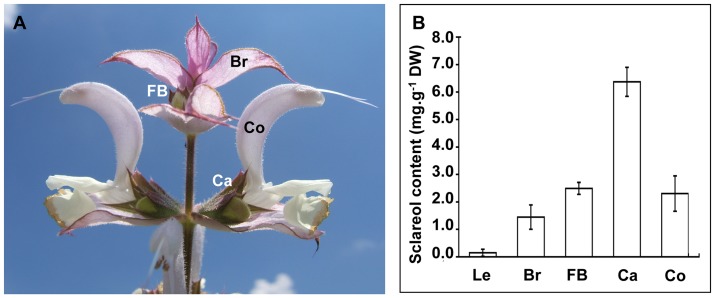
Sclareol contents of mature clary sage inflorescence organs. (A) Fully opened flowers of clary sage with visible calyx, corolla and bract. (B) Sclareol was extracted, analysed and quantified as described under materials and methods on freshly harvested material. Dissected flowers were at a fully opened stage. Each value represents the average value of five independent samples ± CI (α = 0.05). Each sample was a mix of organs originating from three separate plants. Dissected organs were leaves, bracts, calyces (with attached carpels) and corollas (with attached stamens). Legends: Br, bract; Ca, calyx; Co, corolla; FB, flower bud; Le, leaf.

Growers harvest clary sage inflorescences at full bloom stage (figure 1A). These contain individual flowers, their peduncles, bracts and varying quantities of leaves located above the basal rosette and attached to the main inflorescence stem. On an industrial scale, we obtained 100 g of sclareol in 10 kg of essential oil after steam distillation of 1,000 kg of dry inflorescences (*i.e*. 0.01% yield) but 15 kg of sclareol by direct solid/liquid extraction of 1,000 kg of dry straw (*i.e*. 1.5% yield). These results confirm that it is not possible to extract sclareol efficiently by steam distillation of the essential oil. To know if sclareol is lost during the process or if it is not present in the essential oil in the plant, we wanted to localize it more precisely. Indeed, sclareol being a diterpene, it is less volatile than monoterpenes and sesquiterpenes, and it could simply not be efficiently steam-distilled. Thus, in our work, we also include observations *in planta* before and after distillation (see last paragraph of results).

At the laboratory scale, GC-MS analyses of sclareol contents of individual organs revealed that flower calyces were the richest source of sclareol in the plant. These contained 30 times as much sclareol per unit of dry weight as leaves did (figure 1B). Bracts were also a rich source of sclareol (7 times as much as leaves). These data are in agreement with results from Lawrence [4] showing that calyx essential oil is the richest source of sclareol.

Considered together, these data demonstrate that calyces are the prime site of sclareol accumulation in clary sage.

### Observation of trichomes and crystal-like structures on calyx epidermis

**Figure 2:**
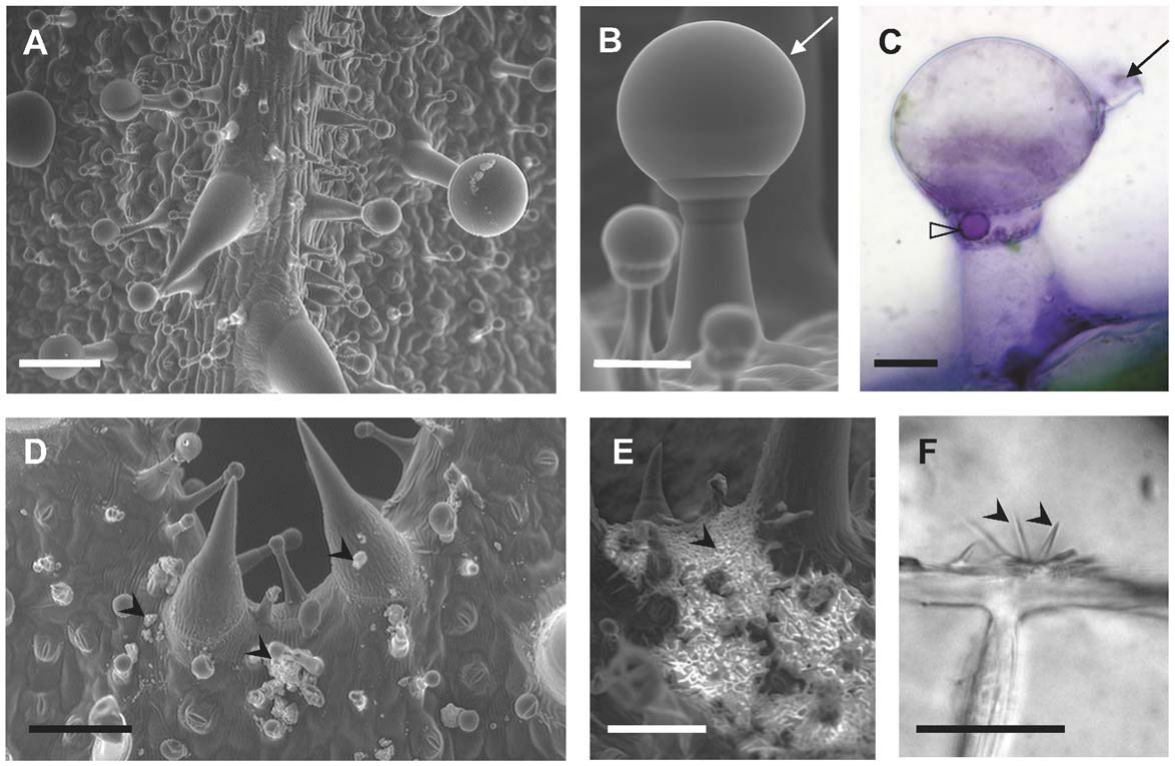
Micrographies of calyx epidermis of clary sage. (A) ESEM view of a calyx epidermis showing the diversity of trichomes. (B) ESEM view of a capitate gland. (C) Nadi staining of a capitate gland with a terpene droplet in the neck cell (hand-made cutting has destroyed the cuticle and led to the loss of essential oil in the subcuticular storage space). (D) ESEM view of epicuticular precipitate-like structures. (E) ESEM view of a cluster of epicuticular crystal-like structures. (F) Light micrography of crystals on the cuticle. Legends: Arrows, cuticle; Full arrowheads, epicuticular crystal and precipitate-like structures; Empty arrowhead, terpene droplet. Scale bars: 100 µm (A, D, E), 50 µm (B), 25 µm (C), 20 µm (F).

An ESEM observation of clary sage calyx epidermis showed that it is densely covered by capitate glands and that peltate glands are rare (figure 2A). These two types of glands were described previously by Werker [5], [6] in this species. We further observed that both gland types accumulate essential oil as in some other essential oil-producing members of the *Lamiaceae*. Our ESEM and light microscopy observations have indeed revealed the existence of a large subcuticular space above the head cells of all mature glandular trichomes (figure 2B). A staining of lipid bodies with Nadi [14] showed the presence of terpenes in large intracellular droplets located within the neck cell above the stalk cell of mature capitate glands (figure 2C).

During ESEM and light microscopy observations, numerous crystals were seen on the surface of the cuticle (figure 2D–F). These were present as clumps of precipitate-like (figure 2D) or crystal-like (figures 2E, F) structures scattered on the entire calyx surface including trichome stems and heads (all types of trichomes) and pavement cells. These structures differed in length, but seemed to have a similar shape and really looked like crystals at high magnification (figure 2F). They were not evenly distributed over the calyx surface but were encountered at random in irregular clusters. They were present on fresh calyces *in natura* since the bud stage, but easier to find in flowers at the fully opened stage. Furthermore, less than one day after harvest, calyces were covered with crystal-like structures. This indicates that precipitation or crystallisation also depends on drying.

### Microscopical analyses of crystal-like structures

**Figure 3:**
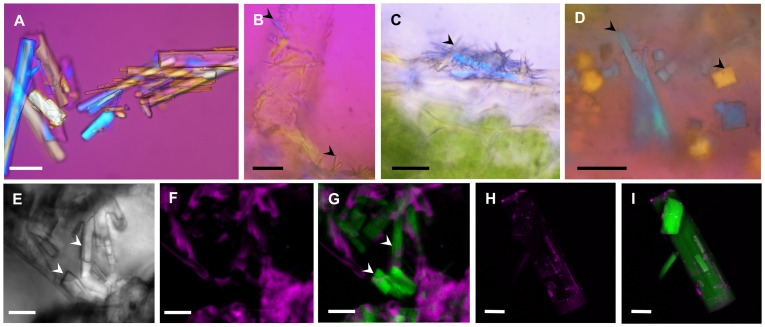
Microscopic imaging of crystal-like structures of clary sage and sclareol crystals. (A) Polarized light micrography of sclareol crystals. (B, C, D) Polarized light micrography of crystal-like structures on the cuticle of calyces showing a cluster on C and different shapes on D. (E) Differential interference contrast micrography of crystal-like structures on the cuticle of calyces. (F) TPF micrography of the same structures as in E. (G) Imaging of SHG signal (green canal) merge with photo F. (H) TPF micrography of sclareol crystals. (G) Imaging of SHG signal (green canal) merge with photo H. Legends: Full arrowheads, epicuticular crystal-like structures. Scale bars: 10 µm (D), 15 µm (B, C, E, F, G), 30 µm (A, H, I).

Since pure sclareol spontaneously crystallises at ambient temperature, we focussed on the crystal-like structures present on the surface of the calyx cuticle. Under polarized light, they displayed the same light refraction pattern as pure sclareol crystals (figure 3A, B). Similar variations in light patterns were generated when the samples were rotated over a 90° angle. This qualitative analysis suggested that pure sclareol and cuticle surface crystals had similar anisotropy and birefringence properties. All cuticle surface crystals observed in clary sage had similar optical birefringence patterns (figure 3C, D).

Under TPF microscopy, clary sage cuticular crystals (observed by DIC in figure 3E) did not emit any light signal (figure 3F). Nevertheless, when SHG patterns were analysed, cuticle surface crystals yielded a signal that could be imaged (figure 3G) and was similar to the one seen with pure sclareol crystals (figures 3H, I; text S1).

Both of these experiments demonstrate that these structures are crystalline and suggest that these crystals are similar to those of pure sclareol.

### Chemical composition of epicuticular crystals

**Figure 4:**
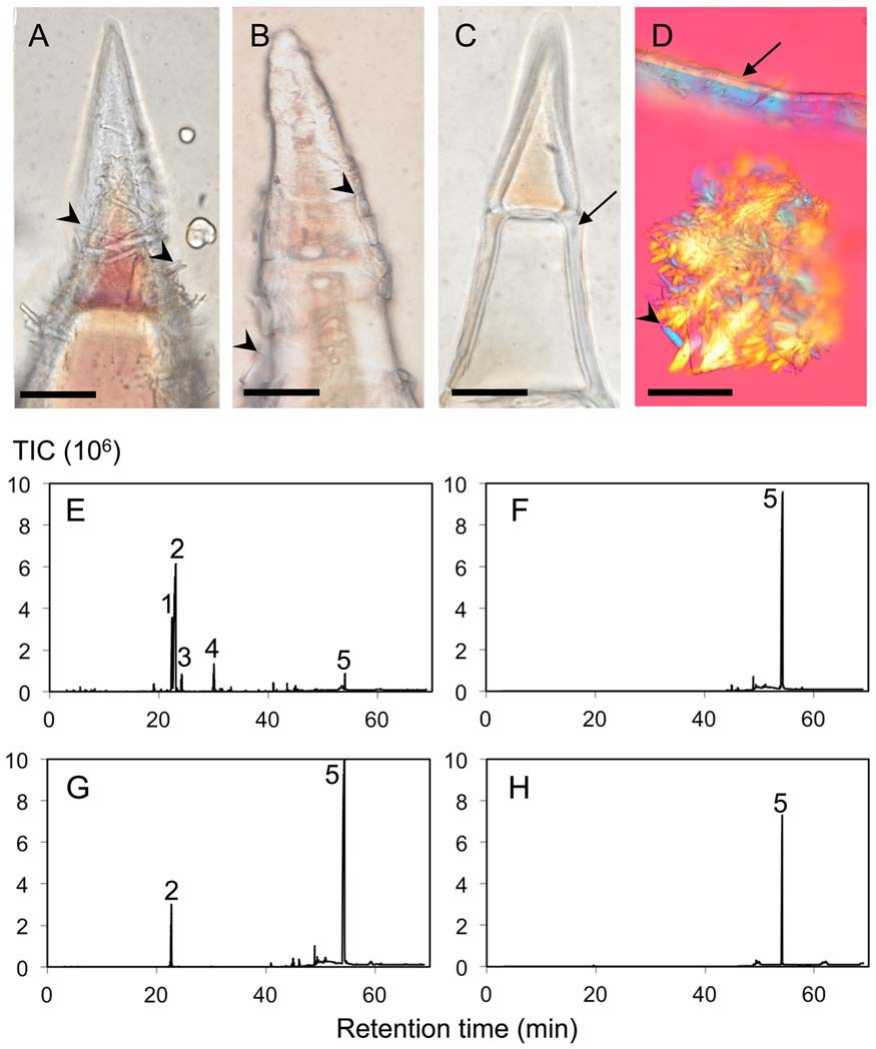
Observation and GC-MS analysis of epicuticular crystals. (A) Light micrography of cuticular crystals just before the industrial process of sclareol extraction. (B) Light micrography of cuticular crystals after steam distillation. (C) Light micrography of the cuticle after solid/liquid extraction with hexane. (D) Polarized micrography of crystals observed in the pellet after centrifugation (note the cuticle scraps). Trichomes were photographed because they allowed the best imaging after the treatments. (E) GC-MS analysis of essential oil obtained by steam distillation of straw and diluted 10-fold in hexane (% of total peak area: 16% peak 1, 62% peak 2, 4% peak 3, 6% peak 4, 2% peak 5). (F) GC-MS analysis of an hexane wash of calyces dissected after steam distillation of the straw (% of total peak area: 97% peak 5). (G) GC-MS analysis of pellets collected after centrifugation of calyces in water and phase extracted with hexane (% of total peak area: 11% peak 2, 84% peak 5). (H) GC-MS analysis of commercial sclareol (purity >2%, 2 g/L in hexane; total peak area, 96% peak 5). Peak identifications are based on retention time, mass spectrum and comparison with authentic standards. Legends: 1, linalool; 2, linalyl acetate; 3, β-caryophyllene; 4, germacrene D; 5, sclareol; Arrows, cuticle; Arrow head, crystals; TIC, total ion count. Scale bars: 30 µm.

In order to confirm that the crystals present on the surface of the calyx cuticle were made of sclareol, their disappearance was followed during the industrial sclareol extraction process (figure 4). As shown on figures 4A and 4B, steam distillation of clary sage inflorescences did not remove the crystals from the cuticle even if plant tissues suffered some deterioration and subcuticular oil pockets had deflated on top of the capitate and peltate secretory trichomes (not shown). This is in agreement with the small quantity of sclareol (2% of the total peak area) found in the essential oil extracts (figure 4E). Conversely, solid/liquid hexane extraction of distilled clary sage inflorescences led to a nearly complete removal of the cuticle surface crystals (figure 4C). A GC-MS analysis of the hexane extract revealed that sclareol was the only major compound of such extract, representing 97% of the total peak area (figure 4F). Numerous substances with MW>160 were also present in the hexane extract, though at much lower levels than sclareol (each one less than 2% of the total extracted matter).

To further confirm the chemical composition of the calyx surface crystals, we attempted to detach the crystals from the cuticle with a paintbrush as previously described [15]. Unfortunately, all of our attempts to obtain pure crystals with this method failed because detached crystal fractions were always heavily contaminated by broken glandular trichomes. These were much larger and brought essential oil compounds with them. When working with fresh material, the long-stalked trichomes glued to the brush while with dry material, all glands spontaneously detached from the calyces upon contact with the brush. A better experimental approach was to centrifuge calyces in water. Crystals were not soluble in water. They formed a deposit in the pellet unlike essential oil that floated. By using this technique, we were able to collect a visible quantity of epicuticular crystals (figure 4D). The crystals were homogeneous in terms of shape under the microscope, and contained few tissue fragments. These crystals dissolved completely in hexane and their GC-MS analysis showed that they were mostly made of sclareol, representing 84% of the total peak area (figure 4G). Linalyl acetate was the major contaminating substance, 11% of the total peak area, and may have originated from co-extracted tissue fragments. As a comparison, an analysis of our sclareol standard is shown (figure 4H). In this experiment, an estimated 500–600 µg of sclareol was extracted from 144 calyces (about 210–220 mg, thus 0.2–0.3% yield).

Taken together, these results demonstrate that calyx epicuticular crystals are made of at least 84% of pure sclareol.

## Discussion

Epicuticular crystals have never been detected in *S. sclarea* [6], [10], [11], [16], [17]. The reason for this lack of detection could be that earlier histochemical studies used bright field microscopy or scanning electron microscopy. In our study, we used ESEM, polarized and SHG microscopy techniques (figures 2, 3; text S1). The advantage of working with ESEM rather than with conventional scanning electron microscopy is that samples are observed without fixation, washing with solvents and metal coating. Such treatments may remove epicuticular crystals from the plant surface. With ESEM, polarized and SHG microscopy techniques, samples can be directly observed without treatment. Furthermore, with bright field microscopy, crystals are very difficult to detect because they are often less than 10 µm long and are totally colourless. With fluorescence microscopy techniques, they cannot be detected because they do not emit fluorescence. Conversely, SHG microscopy is a coherent instantaneous scattering process that uses non-linear contrast and which is particularly adapted to non-centrosymmetric structures like crystals [18]–[22]. It is a technology that has rarely been used in plant biology even though it has already proved to be instrumental during the specific detection of organized structures such as cellulose, starch, stacked membranes and aligned proteins [19], [23], [24] and refs therein.

### In situ sclareol localization

A large body of studies conducted on several *Lamiaceae* species has revealed that terpenes are accumulated in extracellular oil pockets that can break upon aging or contact with an insect [7]. These terpene molecules are synthesized and secreted by head cells of glandular trichomes, and their accumulation takes place under the cuticle. The cuticle is locally so thick that it detaches from the cell wall and later inflates as quantities of deposited oil increase [5], [7]. Such developmental sequences have mostly been demonstrated in the model *Lamiaceae* species, *Mentha* × *piperita*, by analysing the volatile organic compound content of its oil pockets [25], [26], the synthesis of terpenes in detached glands [27]–[29] and the expression pattern and functioning of the enzymes and genes involved in terpene biosynthesis [30]–[33]. Even if the biosynthesis of sclareol in *S. sclarea* is presumably similar to the biosynthesis of terpenes in other *Lamiaceae* species, its end-form accumulation seems, however, to follow a different route. Indeed, our results show that epicuticular crystals are made of relatively pure sclareol. The first argument is that there is a strong correlation between the number of crystals on the epicuticle (figure 4) and the yield of sclareol extraction: crystals are numerous after steam distillation, and the yield of sclareol is then only 0.01%. Conversely, when sclareol is extracted by hexane, the yield reaches 1.5%, crystals are totally absent from the epicuticle after extraction and no other major compound can be detected by GC-MS. The second argument is that crystal pellets (figure 4) are made of 84% of sclareol and only 11% of linalyl acetate. However, pure linalyl acetate is viscous, not crystalline, while pure sclareol is crystalline, with exactly the same optical properties as those of the cuticle crystals (figure 3). Thus, it can be concluded that linalyl acetate is a minor contamination of the crystal pellet by essential oil. Furthermore, it is possible to argue that the sclareol is a contamination of the crystal pellet by essential oil. It would then be impossible to explain why the yield of sclareol is about 0.2 to 0.3% but not 0.01% as the extraction yield obtained with essential oil. It would also be difficult to explain why an unknown compound crystallises exactly like sclareol, *i.e*. in the same crystal shape and in a concentration compatible with the numerous crystals observed on calyces, and why such an unknown compound is dissolved in hexane but not detected by GC-MS.

Schmiderer [34] analysed the essential oil content of clary sage individual glandular trichomes by pressing solid phase microextraction fibres on them. They found a high variability in essential oil composition with sclareol contents ranging from traces to 40–50% of the extracted substances. In agreement, our data suggest that a global steam distillation of clary sage inflorescences empties efficiently glandular trichome essential oil pockets and yields oil that contains a few percentage points of sclareol. Nevertheless, this essential oil-based pool of sclareol only represents a small quantity of the total sclareol content of an inflorescence (0.01%). Most of the sclareol of clary sage inflorescences will be extracted by a subsequent hexane wash (1.5%) by removing the crystals on the plant surface. Furthermore, our work reveals that sclareol is present under a relatively pure form as crystals on the cuticle surface (including trichome cuticle), which seems contradictory with the localization in glandular trichomes made by Schmiderer [34]. Therefore, two hypotheses can be made. Firstly, extracts made with SPME fiber or steam distillation could be contaminated by sclareol crystals (direct contact with the fiber or dissolution by essential oil vapours during distillation). Secondly, sclareol could be secreted by all cell types, and then would pass through the cell wall and the cuticle except for glandular trichomes. Indeed, the essential oil of glandular trichomes is accumulated outside the secretory cells but beneath the thick cuticle [5], [7], [9]. Nevertheless, our results cannot answer such a question, especially because nothing is known about sclareol biosynthesis.

### Epicuticular crystals in plants

Epicuticular crystals have been described in many diverse plant species. They exhibit a great heterogeneity in micro-morphological shapes [35] and their dissimilarities have proved to be phylogenetically informative [35], [36]. In agreement, crystallised substances belong to different classes of chemicals ranging from triterpenoids, phenylpropanoids, sterols and aliphatic derivatives [37]–[39]. To our knowledge, this study is the first report of a diterpene forming epicuticular crystals. Nevertheless, other diterpenes have been detected in cuticle extracts of *N. tabacum* [40], [41], *Pinus radiata* [42] and *Helianthus annuus* [43] where their natural occurrence as epicuticular crystals remains to be assessed.

Epicuticular crystals have been hypothesized to serve various ecological functions. For example, it has been observed that the adhesion of fungal spores to plant surfaces is reduced by the presence of epicuticular crystals [44]. Sclareol, additionally, displays some toxicity against some strains of fungi and bacteria [45]–[49]. Plant-insect interactions are also affected by the physical nature of the cuticle surface. Physical characteristics of crystals may indeed help or impede the locomotion of insects [50] such as in the carnivorous plant *Nepenthes alata*, which produces slippery patches of wax crystals at the entrance of its traps to collect preys [51]. In *Lamiaceae*, flowers are pollinated by insects of the *Apidae* group (*Xylocopa violaceae*) and *Apis sp*. have been seen pollinating field-grown plants during our experiments. Unfortunately, such insects are known to practice nectar larceny by piercing holes in the calyx without pollination benefit for the plant [52]. The very high levels of sclareol crystals on calyces suggest they may play a role in protecting flowers against nectar robbery. Ecological studies of the function of epicuticular sclareol crystals on clary sage calyces would now be of great interest to assess their adaptive value for the plant's fitness.

### Sclareol secretion and crystallisation

The presence of large quantities of sclareol crystals on all parts of the calyx epicuticle raises questions about its biosynthetic origin and crystallisation process. If sclareol was to be synthesized by the underlying cells, the newly synthesized molecules would have to cross the plasma membrane, the cell wall and the cuticle to later crystallize on its surface. In a typical epidermal cell, the cuticle is made of an intracuticular layer with cutin and waxes, and an epicuticular wax layer structured as crystals or films [53]–[55]. Transport mechanisms of waxes and epicuticular compounds through the cell wall and through the intracuticular layer must exist even though they are not completely described [39]. Nevertheless, there is no evidence that sclareol is secreted or not via the same pathway. A passive diffusion or an active transport could be hypothesized. Once substances reach a critical concentration on the cuticle surface, they could undergo phase separation and crystallize spontaneously [38], especially when drying.

Nevertheless, there is a possibility that sclareol is synthesized by head cells of the glandular trichomes, and accumulates in the essential oil pockets that top them. Essential oil could be released by diffusing slowly through the cuticle, like in *Salvia glutinosa* or, more rapidly, by cuticle break, [9] and refs therein. In this case, the flowing of the essential oil along the trichome stems followed by the evaporation of the more volatile constituents would lead to the formation of sclareol crystals on the head-cells, the stalk-cells, and on the pavement cells that surround the glandular trichomes. This latter mechanism has been demonstrated to take place during the production of acyl sugars and duvatrienediol, a diterpene in tobacco leaves [56]. Epidermal peels [57], and more specifically detached glandular trichomes, were shown to synthesize duvatrienediol [58], [59]. A similar mechanism of secretion has been assumed to take place for other diterpenes in tobacco such as *cis*-abienol [60], labdenediol and sclareol [61]. Furthermore, a gene encoding an ATP-binding cassette transporter capable of transporting exogenous sclareol through the plasma membrane [62] has been cloned in *N. plumbaginifolia* [63] and shown to display greater expression in glandular trichomes [64]. Even if this transporter is pleiotropic and is involved in drug resistance, it is possible to argue that it could be involved in a plant producing sclareol naturally.

In the end, the unveiling of the sites of production of sclareol in clary sage will require the deciphering and tissue localisation of its biosynthetic apparatus. The 454 pyrosequencing of calyx cDNAs that has been done recently in clary sage will facilitate the characterization of the biosynthetic enzymes [65].

### Conclusion

In this work, we have shown that pure sclareol crystals have the same optical signature under polarised microscopy and SHG microscopy as crystals naturally present on the surface of the cuticle of *S. sclarea* calyces. Furthermore, hexane wash of steam-distilled *S. sclarea* inflorescences removed most of the calyx surface crystals and sclareol at a yield of 1.5%. A parallel analysis of epicuticular crystals that were detached by centrifugation yielded a similar conclusion. ESEM observations have suggested that sclareol crystals were on the calyx epicuticle. It is concluded that sclareol mainly accumulates as epicuticular crystals in clary sage calyces.

## Materials and Methods

### Materials

Clary sage was grown in fields on the « Plateau de Valensole, Département des Alpes de Haute Provence » (altitude 580 m) under local agronomic practices. Plants were harvested at maximum blooming stage, when more than 50% of the flowers had already opened (figure 2A). Freshly harvested material was either dried or steam-distilled. Solid/liquid extraction was made with hexane. Control sclareol crystals of >99% purity (according to GC-MS analysis respecting AFNOR directives) were obtained by Bontoux S.A. by direct hexane extraction of clary sage followed by several steps of purification. All necessary permits were obtained for the described field studies (Mr JP Pelissier for fields in Banon, and SCA3P and PPV cooperatives for industrial extractions).

### Dissection of plants and terpene extraction

Individual plant parts were collected with scissors except for corollas, which were pulled away with tweezers from the rest of the flower. Stamens are naturally fused to corollas and were not removed prior to analysis. Similarly, calyces were not separated from attached carpels and immature seeds within. Terpenes were extracted by soaking 0.5 g of fresh material overnight at 4°C in 4 mL of hexane containing 20 mg/L camphor as internal standard.

Alternatively, cuticular crystals were collected by placing 144 dry calyces in water and by centrifuging them for 15 min at 12,000×*g*. The supernatant was carefully removed and the pellets re-suspended in water and centrifuged again. Then, the remaining supernatant was removed as well as plant debris. The pellets contained white crystals which were suspended in water (approximately the same volume of water as that of the crystal pellets) to allow their pipetting and pooling in a common glass tube containing hexane (hexane: water, 2∶1). After shaking and phase separation, all crystals had dissolved. The hexane phase was collected and concentrated down to 200 µl under a stream of dry air. This experiment was repeated twice.

#### GC-MS analysis

GC-MS analyses were carried out on an Agilent 6850 GC coupled to a 5973 Agilent MS. A Silica HP-Innowax capillary column (30 m×0.25 mm with a 0.25 µm film – reference 1909N-133E) was used with the injector set at 250°C and 1 mL/min helium as carrier gas. Injection volume was 2 µl with a 10∶1 split ratio. Oven temperature settings were: 2 min, 60°C; 2°C/min ramp to 130°C; 10°C/min ramp to 250°C; 20 min at 250°C. For MS, temperatures of the ion source and GC-MS interface were set at 230°C. Ionizing voltage (ei-mode) was 70 eV and mass scan rate and range were 2.45 scans/s and 35–350 *m/z* respectively. Sclareol identification was based on its retention time (comparison with an authentic sclareol standard) and its mass spectrum (comparison with Wiley, and NIST 05 mass spectra databases). A standard curve of sclareol was built to calculate its concentration.

#### Histochemical staining

Observations were made with a Leitz DMRB microscope with a lambda filter for polarized microscopy. For the Nadi reaction [14], which is often used to localize terpenes in essential oil *in situ*, fresh sections were soaked for 30 min to 1 h in a freshly made mixture of 1-naphtol and *N, N*-dimethyl-*p*-phenylenediamine dihydrochloride according to Bergougnoux [66].

#### ESEM, TPF and SHG imaging

For ESEM imaging, fresh pieces of calyces were laid on a stage fitted to the low-pressure chamber of an S-3000N Hitachi microscope (Tokyo, Japan). To allow observations, samples were cooled from +4°C to a minimum of −20°C by Pelletier effect. Pressure was then set at 110 Pa and tension at 15 kV. For TPF and SHG microscopy, dry pieces of tissues were used on a TCS-SP2 inverted confocal scanning laser microscope (LEICA Microsystems) equipped with DIC technology. The excitation source was a MIRA 900 femtosecond laser from COHERENT powered by a 5W VERDI Nd: YAG continuous laser (Text S1).

## Supporting Information

Text S1 **TPF and SHG microscopy.**
(DOCX)
