# Supplementary material for: Extracellular Localization of the Diterpene Sclareol in Clary Sage (Salvia sclarea L., Lamiaceae)
Source: PLoS One. 2012 Oct 25;7(10):e48253. doi: 10.1371/journal.pone.0048253 (PMC3484996; doi:10.1371/journal.pone.0048253)
Supplement: Text S1 — TPF and SHG microscopy. (DOCX) [file pone.0048253.s001.docx]

**Supporting information:**

***Text S1: TPF and SHG microscopy***

*TPF and SHG methods:* A TCS-SP2 inverted confocal scanning laser microscope (LEICA Microsystems, Germany) equipped with DIC microscopy was used for TPF and SHG imaging. The excitation source was a MIRA 900 femtosecond laser from COHERENT powered by a 5W VERDI Nd:YAG continuous laser. The excitation wavelength was set to 800 nm. The excitation beam was injected in the microscope optical path via a dichroic mirror reflecting light with wavelengths greater than 710 nm. For TPF contrast, the TPF signal was collected backwards through the microscope lens and was sent on the dichroic mirror. The fluorescence spectrum was then spread by a prism and wavelengths ranging from 620 to 680 nm were sent to a photomultiplier tube (PMT). For SHG contrast, the second harmonic signal was collected forward by a condenser lens, passed through an interferential filter (CHROMA HQ400/50, bandpass: 385-425 nm) and finally analysed by another PMT. The responses of both these types of phenomena (TPF and SHG) were non-linear because the fluorescence and second harmonic signals were coming mainly from the focal volume of the excitation beam. This conferred an axial resolution and permitted virtual slicing of the sample.

*Results and conclusion:* The images presented here were obtained by averaging projections of stacks composed of roughly 100 confocal images. The stacks corresponded to a total volume of 63 x 63 x 31 µm^3^ for figure 3G (cuticular crystals) and 138 x 138 x 27 µm^3^ for figure 3I (purified sclareol). As the SHG signal follows quadratically the incident light power and assuming a linearity of the PMT sensitivity as a function of voltage (which is reasonable in the 700-1000 V range that was used in this experiment), the detected signal would be proportional to the squared incident power multiplied by the PMT voltage. When performing such calculation with the incident power and voltage setting values of this experiment, it could be estimated that a similar level of SHG conversion would lead to the same output signal level with a 3% error bracket. Since the grey levels of the images of pure sclareol crystals and cuticle surface crystals differed by less than 3%, both SHG conversion levels could be considered as similar.

*Discussion:* With SHG imaging technology, the incident energy comes from a pulsed laser beam. It is therefore not resonantly absorbed as in TPF microscopy. SHG is a coherent instantaneous scattering process in which two incident photons at a wavelength λ (800 nm in our experiments) interact with crystals to generate a single outgoing photon at exactly λ/2 (400 nm in our case). This latter photon will be in phase with the excitation beam (Chu et al., 2002). This type of non-linear contrast is very specific to non-centrosymmetric structures like crystals and can be easily discriminated from fluorescence signals because of its different origin [18], [20]-[22]. Indeed, the SHG signal is mainly forward-directed because it arises from a coherent generation process, contrary to fluorescence. This is why we used a forward-directed detection scheme. Moreover, the SHG signal is generated precisely at twice the incident frequency and can be specifically selected by the presence of an interferential band-pass filter. Any non-specific fluorescence would begin to significantly contaminate the output signal at wavelengths greater than λ/2 but were rejected by the filter. In our case, SHG signals were detected from pure sclareol crystals and from those present on the surface of the cuticle of *S. sclarea* calyces while no specific fluorescence signal was observed in TPF microscopy.
